# Kidney transplantation using a colon pouch (Mainz pouch III): a case report

**DOI:** 10.3325/cmj.2019.60.545

**Published:** 2019-12

**Authors:** Dean Markić, Romano Oguić, Kristian Krpina, Antun Gršković, Ivan Vukelić, Sanjin Rački, Aldo Ivančić, Davor Primc, Josip Španjol

**Affiliations:** 1 Department of Urology, University Hospital Rijeka, Rijeka, Croatia; 2 Faculty of Medicine, University of Rijeka, Rijeka, Croatia; 3 Department of Nephrology, Dialysis and Transplantation, University Hospital Rijeka, Rijeka, Croatia; 4 Department of Surgery, University Hospital Rijeka, Rijeka, Croatia

## Abstract

Kidney transplantation is the most efficient method of renal replacement therapy. When this method is performed, native urinary bladder is the preferred urinary reservoir. However, in some patients with an anatomically and functionally abnormal lower urinary tract, the urinary bladder cannot be used for transplantation. In these patients, urinary diversion should be performed before kidney transplantation. We present a case of a 32-year-old male patient with orthotopic kidney transplantation performed using a colon pouch (Mainz-pouch III). He was born with severe anomalies including sacral agenesis, anorectal atresia, and hypospadias, which were corrected during childhood. Neurogenic bladder with severe vesicoureteral reflux led to end-stage renal disease. This dysfunctional bladder was unsuitable for kidney transplantation, and a staged approach for future transplantation was chosen. The first step was the creation of urinary diversion. Due to a short appendix, we created a continent, colon pouch (Mainz pouch III). Two years later, orthotopic kidney transplantation was performed using a right cadaveric kidney. The renal vessels were anastomosed to the aorta and inferior vena cava and the pyelon to the native ureter. Four years after transplantation, the patient has stable renal function without any complications. This is the first documented case of using Mainz-pouch III as a reliable option for kidney transplantation in selected patients.

At the beginning of the era of kidney transplantation (KT), a normal urinary bladder was considered an indispensable condition. Over time, the patients with dysfunctional urinary bladders became candidates for transplantation. The first KT using an ileal conduit in a patient with a severely damaged urinary bladder was done in 1966 (1). Since then, the number of KTs in patients with abnormal lower urinary tracts has gradually increased.

Up to 15% of patients with end-stage renal disease (ESRD) have abnormalities of the lower urinary system, which can lead to kidney deterioration (2). In the pediatric population with ESRD, this rate increases up to 30% (3). In patients with a dysfunctional bladder (small volume, poorly compliant bladder), transplantation must be avoided because the graft will be damaged by the same mechanisms as those that damaged native kidneys. In these patients, it is recommended to perform pretransplant surgical correction of the urinary tract before KT. The urinary bladder can be augmented with a segment of the ileum, colon, or stomach, with the intention to create a bladder with low pressure, enough capacity, and adequate compliance to protect the upper urinary tract and renal allograft. The other possibility is to create a urinary diversion, with or without a cystectomy.

Urinary diversion is defined as the redirection of urine flow from its physiological route as a consequence of a diseased or defective ureter, bladder, or urethra. In most cases, this diversion creates a route that leads to the skin (urostomy), but it could also lead to other systems (rectum, sigmoid colon). Some of them have a continent mechanism (Mainz pouch I), and others do not (ileal conduit). Definitive urinary diversion includes ureterosigmoidostomy (Mainz pouch II), incontinent cutaneous urinary diversion (ileal conduit), and continent cutaneous urinary diversion (Indiana pouch, Kock pouch, Mainz pouch I, Mainz pouch III).

We present a patient from our transplant center in whom orthotopic kidney transplantation was performed using a colon pouch (Mainz-pouch III). This is the first described case of such transplantation in the literature.

## CASE REPORT

The patient was born with significant congenital abnormalities (sacral agenesis, anorectal atresia, and hypospadias) (Table 1). Immediately after birth, a permanent colostomy was performed, with revision when he was 18 months old. His congenital abnormalities resulted in neurological dysfunction of the lower urinary tract with neurogenic bladder, bilateral vesicoureteral reflux, and bilateral megaureters. To prevent the deterioration of kidney function, ureterocutanostomy was performed. The left ureter was terminolaterally anastomosed to the right ureter (transureteroureterostomy), and the right ureter was diverted to the skin (urostomy). At the age of five, nephrotic syndrome was diagnosed, and the kidney biopsy revealed chronic membranous glomerulonephritis. He received corticosteroids, cyclophosphamide, cyclosporine, and azathioprine, but his chronic kidney disease progressed to ESRD. In January 2012, at the age of 30, the patient started hemodialysis. In the same year, he presented to our transplant center, which is a referral center for kidney transplantation in Croatia. The multidisciplinary team decided that he was an appropriate candidate for orthotopic kidney transplantation with urinary diversion and used a staged approach. Since the patient was working and socially active, we planned to construct a continent reservoir.

**Table 1:** Medical history timeline

Year/age	Diagnosis	Therapeutic intervention
1982/0 months	Sacral agenesis, anorectal atresia, hypospadias	Permanent colostomy
1983/18 months	Stenosis of colostomy	Revision of colostomy
1984/24 months	Neurogenic bladder, bilateral vesicoureteral reflux, bilateral megaureters	Ureterocutanostomy
1987/5 years	Chronic kidney disease (biopsy proven chronic membranous glomerulonephritis)	Drug therapy (corticosteroids, cyclophosphamide, cyclosporine, azathioprine
2012/30 years	End-stage renal disease	Hemodialysis
2012/30 years	Preparation for transplantation including the management of recurrent left sided pyelonephritis	Simultaneous left nephrectomy, creation of urinary diversion: Mainz pouch III, ureterocutanostomy removal, implantation of right ureter in pouch
2014/32 years	End-stage renal disease	Orthotopic right kidney transplantation using Mainz pouch III as urinary diversion

**Figure 1:**
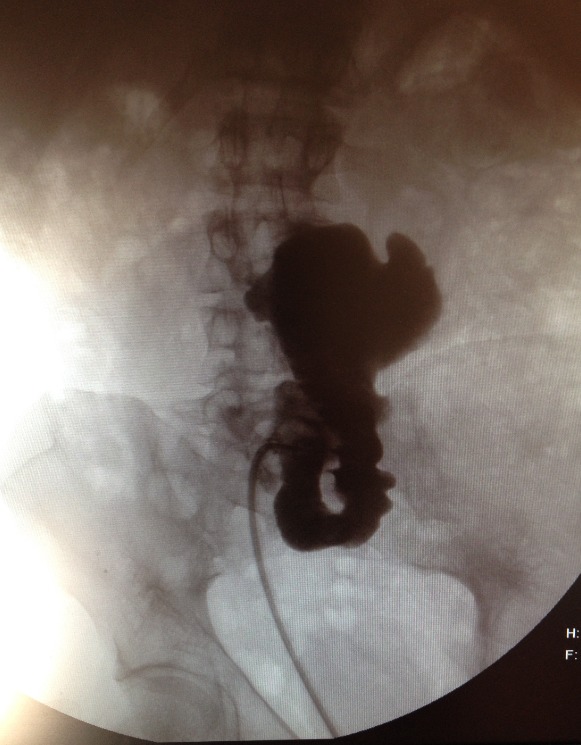
Pouchography after the creation of a reservoir.

The first operation was performed in November 2012 using a midline laparotomy approach. Because of recurrent left sided pyelonephritis, we removed the left kidney along with the ureter and removed urostomy. The right kidney and right ureter were preserved. Another part of this operation was the creation of urinary diversion for future transplantation. The small and large bowel were mobilized carefully. Preoperatively, we planned to create an ileocolonic reservoir (Mainz pouch I); however the appendix, which serves as the connection between the skin and the pouch, was too short. Therefore, we decided to perform another type of urinary diversion, Mainz pouch III. Mainz pouch III is a colonic, continent reservoir. We isolated a part of the ascending and transverse colon (approximately 35 cm) for the creation of a reservoir with adequate capacity. The operation was performed as initially described by Leissner et al (4). The right ureter was implanted in the pouch using an antireflux technique. The urinary catheter was inserted into the pouch through the umbilicus because in this type of operation the umbilicus serves as urostomy. Postoperatively, the patient developed paralytic ileus, which resolved with prokinetic drugs. The patient was instructed to irrigate the pouch regularly, twice a week, since only the right kidney produced a scarce amount of urine. This was necessary due to bowel mucous production and to keep the pouch from constricting. The postoperative capacity of the pouch was more than 200 mL (Figure 1). Approximately two months after the operation, the urinary catheter was removed, and the patient continued with self-catheterization. At the end of this first stage, the patient had a continent pouch with a catheterizable stoma prepared for right orthotopic kidney transplantation.

**Figure 2:**
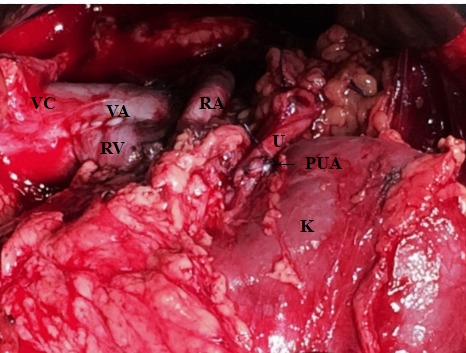
Intraoperative view of orthotopic kidney transplantation in the right lumbar region. K – kidney; VC – vena cava; RV – renal vein; RA – renal artery (crossing vena cava anteriorly); U – ureter; VA – venous anastomosis between the vena cava and renal vein; PUA – pyeloureteral anastomosis.

In November 2014, through the Eurotransplant allocation system we received an offer for a left cadaveric kidney. The donor was a 45-year-old male with brain death resulting from a traumatic bilateral subdural hematoma. The HLA matching was as follows: HLA-A (1 match), HLA-B (0), HLA-Cw (1), HLA-DR (1), and HLA-DQ (1). The crossmatch was negative. Since orthotopic kidney transplantation was planned, the surgical approach was a right sided lumbotomy over the 11th rib extending to the lateral edge of the rectus muscle. The complete operation was performed extraperitoneally. Right sided nephrectomy was done. The ureter was ligated at the level of the pyeloureteral junction. Intraoperatively it was observed that the artery and vein of the right kidney were hypotrophic and unsuitable for vascular anastomosis. This is why we prepared the inferior vena cava and aorta for this purpose. On a back-table, the left cadaveric kidney with one renal artery, vein, and ureter was prepared. The cadaveric kidney was placed in the operative field with the renal pelvis as the most superficial part of the hilar structures. First, we anastomosed the renal vein with the inferior vena cava and the renal artery with the aorta using running, nonabsorbable, monofilament 5-0 polypropylene sutures. The duration of vascular anastomosis was 40 minutes. After declamping the renal vessels, the graft immediately became well vascularized with normal consistency. Finally, we anastomosed the renal pelvis of the transplanted kidney and the right native ureter (pyeloureteral anastomosis) using monofilament, absorbable, 5-0 polydioxanone sutures (Figure 2). Urinary anastomosis was protected using JJ endoprothesis (26 cm long, diameter 6 Ch). To prevent the sliding of the transplanted kidney, nephropexy was performed. The overall operation time was 330 minutes, and cold ischemia time was 15 hours. The diuresis level on the first day was 1900 mL. For the first three postoperative days, the patient received heparin intravenously (30.000 IU/24 hours), followed by low molecular weight heparin. The drain was removed on the ninth day. The patient had a positive urine culture: *Escherichia coli* and *Proteus mirabilis,* more than 100 000/mL, both sensitive to ampicillin, piperacillin + tazobactam, cefuroxime, and gentamicin. A combination of piperacillin and tazobactam was administered (2 × 4.5 g intravenously) for 14 days, followed by cotrimoxazole prophylaxis (450 mg once daily) for six months. From the time of operation to the present, immunosuppressive protocol consisted of corticosteroids, mycophenolate mofetil, and tacrolimus. Twenty days after the operation, the patient was discharged from the hospital with excellent kidney function: serum urea 6.3 mmol/L and serum creatinine 103 μmol/L.

**Figure 3:**
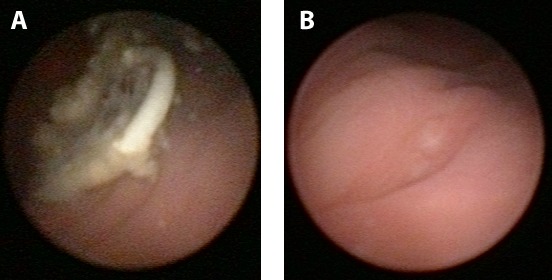
Endoscopic view of the colon pouch with ureteral endoprothesis exiting from the ureteral orifice and partly embedded in mucus (**A**). Ureteral orifice after removal of the endoprothesis (**B**).

In January 2015 (two months after the operation), the patient was admitted to the hospital for the removal of the JJ endoprothesis. During general anesthesia, we entered the pouch through the urostomy site in the umbilicus using a semirigid ureteroscope. Some mucus was found in the pouch and was removed. The protected anastomosis between the ureter and the pouch was visualized, and the endoprothesis was grasped and removed (Figure 3). One week after the stent removal, control ultrasonography revealed a well vascularized graft without hydronephrosis. Since then, the patient has been regularly followed. He empties his pouch with intermittent self-catheterization 4-6 times in a 24-hour period. Currently, four years after transplantation, the patient is without surgical, immunological, or infective complications. The graft function is stable. In 2016, he became the father of fraternal twins with the help of assisted reproductive technology.

## DISCUSSION

Currently the most common type of urinary diversion in the kidney transplant population is the ileal conduit (or Bricker operation). The use of a colon conduit was first described by Tunner et al in 1971 (5). Over time, these procedures have increased in number and type, but the overall number of KTs using urinary diversion is low.

The study with the largest number of transplanted patients with urinary diversion included 54 patients with 59 renal transplants (6). All patients had an ileal conduit. The median patient age at the time of transplantation was 29.3 years. Graft survival at 1, 5, 10, and 15 years was 90%, 63%, 52%, and 52%, respectively. Patient survival was 95%, 83%, 69%, and 69%, respectively. Patients had many complications (especially urinary tract infections and complications related to the ileal conduit), but long-term graft and patient survival was comparable to that in the normal transplant population (6).

The experience of using the large bowel for urinary diversion in KT is very limited. In Table 2, we present the literature data about KT using a colon pouch, conduit, or neobladder (5,7-10). Kocot et al (10) presented their long-term results of 18 kidney transplant patients with different types of continent urinary diversion, in 3 of whom the colon was used. KT was performed 4-156 months after the creation of continent urinary diversion. Day and night continence was achieved in all patients with cutaneous urinary diversion and orthotopic substitution. Five patients (27.8%) were diagnosed with acute pyelonephritis rather than having bacterial colonization in all reservoirs. Kocot et al did not observe stenosis of the ureterointestinal anastomosis, although this is found in up to 6.5% of nontransplant patients (11). They found complications of continence mechanisms requiring surgical revision in 18.7% of patients, which is similar to the results of other studies (11). The second transplantation was performed in two patients with thrombosis of the renal vein and chronic graft dysfunction and two more patients resumed hemodialysis. In the remaining patients, with a mean follow-up of 90 months, kidney function was stable (10).

**Table 2:** Kidney transplantations using a colon pouch, conduit, or neobladder*

Author	N	Urinary diversion	Age (years)	Cause of ESRD	Graft survival (months)	Patient survival (months)	Renal function (creatinine in μmol/L)	Complications
Tunner (5)	1	sigmoid colon conduit	11	posterior urethral valves	unknown	unknown	123	unknown
Riedmiller (7)	1	sigmoid neobladder†	50	contracted bladder (tuberculosis)	52	52	150	unknown
	1	colon pouch†	30	myelomeningocele	32	32	115	unknown
Rigamonti (8)	2	sigmoid colon conduit	17 20	neurogenic bladder neurogenic bladder	184 148	184 148	114 142	UTIs no
Ishida (9)	1	right colon pouch (Indiana)	32	neurogenic bladder	12	12	normal	no
Kocot (10)	1	sigmoid neobladder†	50	contracted bladder (tuberculosis)	141	141	141	unknown
	1	left colon pouch†	30	myelomeningocele	128	128	176	unknown
	1	sigmoid neobladder	50	bladder TCC	13	13	106	unknown

*ESRD – end-stage renal disease; UTIs – urinary tract infections; TCC – transitional cell carcinoma.

†Same patients in different articles.

Mainz pouch III as a continent, colonic pouch was first introduced in 2000 (4). An experienced medical team from Mainz used this type of urinary diversion in 44 female patients with gynecological tumors after pelvic irradiation to avoid the use of irradiated bowel segments. There were no early complications, while late complications included incontinence in 2 (4.5%) patients (resolved with the creation of a new efferent segment) and stoma stenosis in 6 (13.6%) (4 treated with endoscopic incision and 2 underwent YV plastic). These satisfactory long-term results promoted this operation as a method of choice in patients with previous pelvic irradiation (4). Another study included 24 patients (6 nonirradiated patients) with Mainz pouch III continent urinary diversion (12). Twenty-two of these patients were treated because of malignancy (gynecological tumors, rhabdomyosarcoma of the prostate, bladder cancer) and 2 because of a benign disease (neurogenic bladder). The follow-up lasted up to 65 months. Twenty patients achieved complete continence (83.3%) and 4 (16.7%) used a protective pad between intermittent self-catheterization. The only pouch-related complication (outlet stenosis) was found in one patient (4.2%) and was surgically corrected.

In our center, we also started to use Mainz pouch III for urinary diversion in patients with gynecological tumors and after pelvic irradiation. This valuable experience allowed us to use this procedure as pretransplant preparation for our kidney transplant recipients. We want to point out that such operations should be performed in centers with adequate experience, especially in cases when, instead of one demanding operation, an intraoperative finding necessitates the use of a different, even more demanding, operation.

The patients who undergo this type of urinary diversion must be informed about the risks of the procedure, including the risks associated with self-catheterization, and should be assessed for compliance. Self-catheterization is mandatory in continent cutaneous diversion, and most authors agree that, although potentially complicated by chronic bacteriuria, it is safe for immunosuppressed transplant patients (10,13). The contraindications for this type of urinary diversion are colon impairment (colorectal cancer, stenosis, diverticular disease, short bowel syndrome) and inadequate compliance (14). The follow-up by urologists includes standard surveillance with determination of acid-base balance and stoma-related problems (incontinence, impaired catheterization) (10). After 3 years of follow-up, it is advisable to perform a yearly pouch endoscopy for malignancy screening (10).

This technique has some advantages compared with ileal or ileocolonic urinary diversion: preservation of the ileocecal valve (without negative influence on stool frequency and enterohepatic circulation), normal intake of vitamin B12, and normal bile acids absorption (12,14). Another advantage of Mainz pouch III is that the efferent limb is created with use of the large bowel instead of the small bowel. The large bowel has a wider lumen than the appendix (frequently used in the other types of pouches), allowing the use of a larger catheter. Additionally, it is easier to flush the reservoir to remove mucus and to perform pouchoscopy of the large bowel, if needed, using both pediatric and adult instruments (12). The main disadvantage is that the operation is relatively complicated compared with the creation of a simple ileal conduit. Additionally, urologists are much more familiar with the use of an ileal segment than the large bowel.

The population of KT patients with urinary diversion is small, and their outcomes are difficult to compare due to different types of operations. The 5-year graft survival is up to 78% and 15-year graft survival is up to 69% (6,8,15-17).

The use of bowel segments in urinary diversion in KT patients is associated with an increased incidence of urinary infections, metabolic changes, and possible tumor development. A few studies reported that chronic bacteriuria or urinary tract infections (UTIs) did not lead to graft loss, but the incidence of UTIs was high (15,17,18). In other studies, severe UTIs were responsible for graft loss in KT patients with urinary diversion (19,20). The most prevalent opinion today is that urinary diversion significantly increases morbidity in KT patients but does not affect graft and patient outcome (21).

Different studies show different prevalence of metabolic changes – from no changes to 85% (8,18,22). The most prevalent type of metabolic imbalance is metabolic acidosis (23-25). Therefore, these patients need to be regularly checked for acid-base balance.

The development of neoplasms, mainly due to chronic immunosuppression, is a well-known possible long-term complication of organ transplantation. Primary neoplasms can be found in urinary diversion in nontransplanted patients (26). There is only one reported case of primary adenocarcinoma of an ileal conduit in a renal transplant patient, which manifested 19 years after KT (27). It has not been elucidated yet which is the proper screening protocol after KT with urinary diversion because of limited data but it could be a combination of urinalysis, urine cytology, imaging techniques, and pouch endoscopy (10,27).

In conclusion, the native bladder is the most efficient reservoir used for KT. Patients with severe anatomical or functional abnormalities of the lower urinary tract require urinary diversion before transplantation. Mainz-pouch III, as a colon continent pouch, can be used as a reliable type of urinary diversion in selected transplant patients.
